# Homozygous deletion in *MICU1* presenting with fatigue and lethargy in childhood

**DOI:** 10.1212/NXG.0000000000000059

**Published:** 2016-03-03

**Authors:** David Lewis-Smith, Kimberli J. Kamer, Helen Griffin, Anne-Marie Childs, Karen Pysden, Denis Titov, Jennifer Duff, Angela Pyle, Robert W. Taylor, Patrick Yu-Wai-Man, Venkateswaran Ramesh, Rita Horvath, Vamsi K. Mootha, Patrick F. Chinnery

**Affiliations:** From the Wellcome Trust Centre for Mitochondrial Research (D.L.-S., H.G., J.D., A.P., R.W.T., P.Y.-W.-M., R.H., P.F.C.), Institute of Genetic Medicine (D.L.-S., H.G., J.D., A.P., P.Y.-W.-M., R.H.), and Institute of Neuroscience (R.W.T.), Newcastle University, Newcastle upon Tyne, United Kingdom; Howard Hughes Medical Institute (K.J.K., D.T., V.K.M.), Department of Molecular Biology, Massachusetts General Hospital, Boston, MA; Department of Paediatric Neurology (A.-M.C., K.P.), The General Infirmary, Leeds, United Kingdom; Department of Child Neurology (V.R.), The Royal Victoria Infirmary, Newcastle upon Tyne Hospitals NHS Trust, Newcastle upon Tyne, United Kingdom; Department of Systems Biology (V.K.M.), Harvard Medical School, Boston, MA; Broad Institute (V.K.M.), Cambridge, MA; Department of Clinical Neurosciences (P.F.C.), University of Cambridge; and MRC Mitochondrial Biology Unit (P.F.C.), Cambridge Biomedical Campus, United Kingdom.

## Abstract

**Objective:**

To define the mechanism responsible for fatigue, lethargy, and weakness in 2 cousins who had a normal muscle biopsy.

**Methods:**

Exome sequencing, long-range PCR, and Sanger sequencing to identify the pathogenic mutation. Functional analysis in the patient fibroblasts included oxygen consumption measurements, extracellular acidification studies, Western blotting, and calcium imaging, followed by overexpression of the wild-type protein.

**Results:**

Analysis of the exome sequencing depth revealed a homozygous deletion of exon 1 of *MICU1* within a 2,755-base pair deletion. No MICU1 protein was detected in patient fibroblasts, which had impaired mitochondrial calcium uptake that was rescued through the overexpression of the wild-type allele.

**Conclusions:**

*MICU1* mutations cause fatigue and lethargy in patients with normal mitochondrial enzyme activities in muscle. The fluctuating clinical course is likely mediated through the mitochondrial calcium uniporter, which is regulated by MICU1.

Mitochondrial disorders can present with a multisystem neuromuscular disorder or can affect only one organ system, such as skeletal muscle, where fatigue and subjective muscle weakness may be the only symptoms. Although molecular genetic testing can reveal the diagnosis in some patients, clinical evaluation often involves a muscle biopsy followed by the biochemical analysis of mitochondrial respiratory chain enzymes. Here we describe 2 cousins with normal mitochondrial electron transport chain enzyme activities who had a homozygous deletion of exon 1 of *MICU1* that was detected by analyzing the depth of exome sequence coverage. Functional studies revealed a defect of mitochondrial calcium handling, providing an explanation for their fluctuating clinical course.

## METHODS

### Patients.

**Figure 1:**
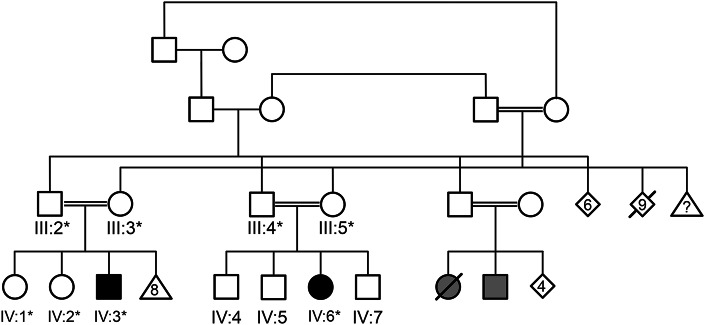
Pedigree of the family described in the case report IV:3 is the index case and IV:6 is her cousin. DNA was available for family members indicated with an asterisk, allowing the segregation analysis shown in figure 2. The 2 gray-shaded individuals have not been assessed in the United Kingdom: 1 failed to walk and died in infancy and the other is reported to have muscle problems.

A 9-year-old girl was referred in 2011 with 4 years of episodic fatigue and lethargy causing frequent school absences. The third child of healthy parents, her in utero and psychomotor development was normal (figure 1). Initially the episodes accompanied minor viral infections and evolved over hours. She would become pale and sweaty and then lethargic and sleepy. At times she was unable to stand unaided and became noncommunicative and unrousable. Now she is 13 years of age and the episodes are precipitated by minimal exercise, such as running down the road. Avoiding physical activity has reduced the frequency of the “attacks,” but after 100 m of walking she now develops muscle aches that limit her activities and resolve after 15 minutes of rest. She is in the 2nd percentile for weight and the 9th percentile for height; her muscles are thin but strong between the episodes. There are no neurologic or ophthalmologic signs. The nonspecific episodes were not investigated until her serum creatine kinase (CK) was measured (497 IU/L between attacks and 2,067 IU/L during attacks). Other blood tests, including lactate, urine organic and amino acids, and acylcarnitines, were normal.

Her cousin described similar episodes on a more complex background. After a normal pregnancy and birth, delayed development was noted at 6 months when nystagmus and an abnormal red reflex revealed cataracts. In early childhood he had episodes of clumsiness with falls associated with intercurrent illness accompanied by headaches and vomiting. Now 12 years of age, he has frequent classic migraines and develops muscle aches after 15 minutes of light exercise. This is associated with intense lethargy, poor concentration, and occasional confusion, which resolve spontaneously within hours or days. Decreasing physical activity to a bare minimum has reduced the frequency of the attacks. He is in the 50th percentile for height and weight and has low-set big ears, a prominent chin, and long thin fingers. He has mild learning difficulties, pendular nystagmus, bilateral optic atrophy, mild hypotonia, and global muscle weakness leading to a positive Gower maneuver. Normal blood tests included glucose and lactate levels between and during the attacks, urine organic and amino acids, and acylcarnitine profile. CK levels >2,000 IU/L have been noted. ECG, echocardiography, and brain MRI were normal. Muscle biopsy revealed rare atrophic fibers, increased internal nuclei, normal mitochondrial respiratory chain complex activities, normal mitochondrial DNA levels, and normal electron microscopy. The karyotype and array comparative genomic hybridization were normal.

### Molecular genetics.

Blood genomic DNA was fragmented from both affected cousins (IV:3 and IV:6), exome-enriched, and sequenced (Illumina TruSeq 62 Mb exome capture and HiSeq 2000, 100 bp paired-end reads). In-house bioinformatic analysis included alignment to UCSC hg19 and using Burrows-Wheeler Aligner and Genome Analysis Toolkit (GATK) to detect single nucleotide variants (SNVs) and small insertion/deletions across all samples using standard filtering parameters according to GATK Best Practice Recommendations.^1^ We sought rare, predicted protein-altering homozygous and compound heterozygous variants that were shared between the 2 affected cousins with minor allele frequency (MAF) <0.005 in the ExAC and NHLBI-ESP6500 databases, MAF <0.02 in the CG69 database, and MAF <0.01 in 337 unrelated in-house controls.^2–4^ Copy number variant analysis was performed using ExomeDepth.^5^ Multiple primer pairs were designed to define the deletion breakpoint using long-range PCR (LA-Taq). Sanger sequencing was used to define the breakpoint. Breakpoint-specific primers were used to track the deletion in the family.

### Cell biochemistry.

Primary skin fibroblasts were derived from both patients (IV:3 and IV:6) and a heterozygous relative (III:3). Oxygen consumption, extracellular acidification studies, Western blotting, and calcium imaging were performed as described.^6–8^

### Standard protocol approvals, registrations, and patient consents.

This study had national ethical review board approval and local institutional approval. Informed consent was provided by the participating families.

## RESULTS

**Figure 2:**
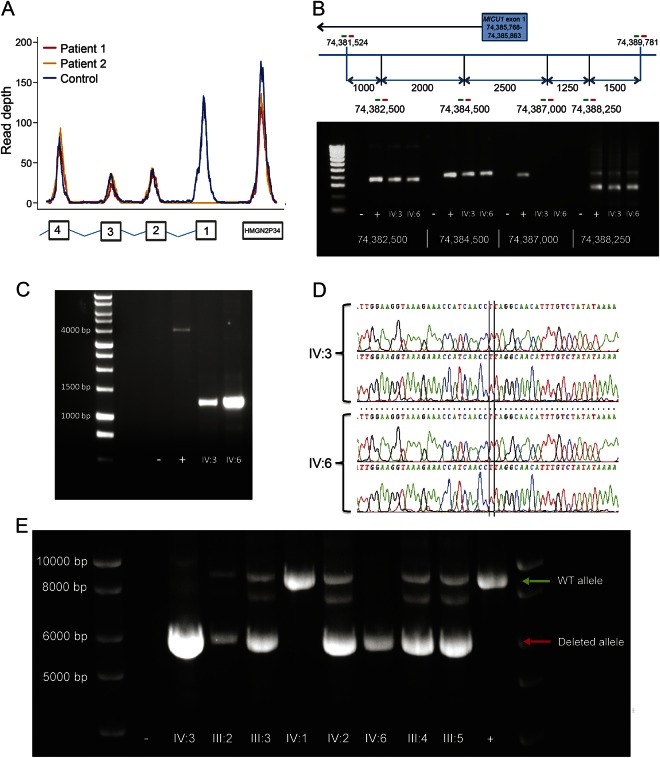
Identification of the deletion (A) Exome read depth across *MICU1* for the 2 patients (IV:3 = patient 1, red; IV:6 = patient 2, yellow) and a representative control (blue) showing a likely homozygous deletion involving exon 1 in IV:3 and IV:6. (B) Long-range PCR primer walking demonstrated the limits of the deletion within the region identified through analysis of exome sequencing coverage (oligonucleotide primers shown as red and green bars). PCR amplification using the named primer pairs shows absence of a product with combination 74,387,000. − = no DNA control, + = healthy control. (C) Long-range PCR using the forward primer from pair 74,384,500 and the reverse primer from pair 74,388,250 amplified a ∼1-kb fragment from the 2 affected cousins, which was small enough for Sanger sequencing. Primers: Fwd TTCCCTTTCTCCTCAGGCAC, Rev GTCTACCGGATTCAGGCGAT. When compared with control DNA, this equated to a ∼2.7-kb homozygous deletion in the patients. (D) Sanger sequence from the 2 affected cousins (IV:3 and IV:6) showing the homozygous deletion breakpoint. Primers: Fwd TTCCCTTTCTCCTCAGGCAC, Rev GTCTACCGGATTCAGGCGAT. (E) Segregation analysis of the *MICU1* deletion. PCR spanning the breakpoint showing the homozygous deletion in the 2 affected cousins (IV:3 and IV:6) and a heterozygous deletion in other family members (III:2, III:3, III:4, III:5, and IV:2). Primers: Fwd CCTGGGCGACAAGTGTAAAA Position Chr 10:74,381,222; Rev CCCAGGCATTTGATCACCAG Position Chr 10:74,390,095. Amplification of the mutant allele in heterozygous carriers of the *MICU1* deletion produced an additional intermediate band of ∼8 kb that is unexplained but did not affect the segregation analysis. Other symbols refer to the pedigree shown in figure 1. WT = wild type.

**Figure 3:**
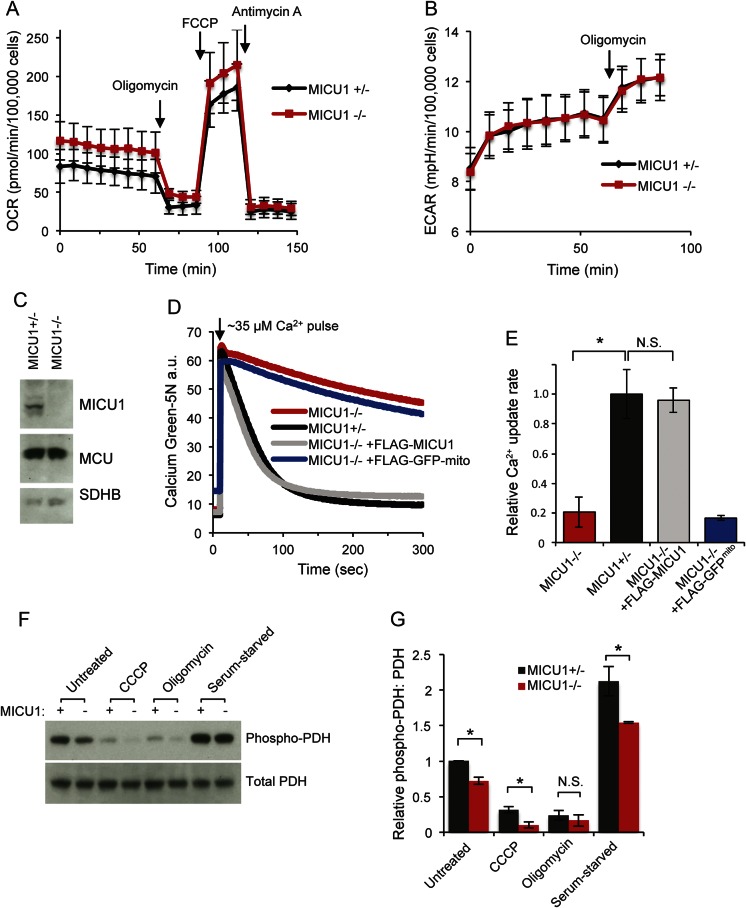
Loss of MICU1 leads to a deficit in mitochondrial calcium handling without impairing respiration (A) Oxygen consumption rates (OCR) and (B) extracellular acidification rates (ECAR) of MICU1^+/−^ and MICU1^−/−^ fibroblasts were measured by Seahorse XF analysis. Mean values ± SE (error bars) from 3 independent experiments are shown (differences are not statistically significant). (C) Immunoblot of whole cell lysates from MICU1^+/−^ and MICU1^−/−^ fibroblasts. (D) Digitonin-permeabilized fibroblasts were given a pulse of 35 μM CaCl_2_ while monitoring extramitochondrial Ca^2+^ with calcium green-5N.^8^ (E) Quantification of the rate of calcium uptake from calcium uptake traces including those in D (using linear fit from 30 to 40 seconds). Mean values ± SD (error bars) from 3 independent experiments are shown. (F) MICU1^+/−^ (+) and MICU1^−/−^ (−) fibroblasts were exposed to different conditions (untreated, 500 nM CCCP, 1 μM oligomycin, or serum starvation) and pyruvate dehydrogenase (PDH) and phosphorylated PDH (at S293) were measured by immunoblot after lysing the cells in 1% Triton-X-100, 150 mM NaCl, 25 mM Hepes pH 7.4, and protease and phosphatase inhibitors. (G) Quantification of 3 independent experiments including the representative experiment in F are shown. Mean values ± SE (error bars). MCU = mitochondrial Ca^2+^ uniporter; SDHB = succinate dehydrogenase subunit B.

No plausible pathogenic SNVs were identified in the exome sequence (table e-1 at Neurology.org/ng). Analysis of exome coverage revealed a likely homozygous partial deletion of *MICU1* (figure 2A), which was mapped by long-range PCR (figure 2, B and C). Sanger sequencing defined the breakpoint (Chr 10: 74,385,085-74,387,860, figure 2D) and the size of the deletion (2,775 nucleotide pairs). Breakpoint primers were used to track the *MICU1* deletion in family members (figure 2E), which showed complete segregation with the disorder. Fibroblasts from patients respired normally, showing oxygen consumption and extracellular acidification rates similar to the heterozygous fibroblasts (figure 3, A and B). MICU1 protein was not detectable by immunoblot in the patient fibroblasts, but mitochondrial Ca(2+) uniporter protein levels were normal (figure 3C). The cells lacking MICU1 showed impaired mitochondrial calcium uptake, which was rescued by introducing exogenous MICU1 but not a control mitochondrial-targeted green fluorescent protein (figure 3, D and E).

## DISCUSSION

The homozygous *MICU1* deletion is highly likely to be responsible for the disorder affecting the 2 cousins because (1) the mutation has not been described before in large control exome databases; (2) it showed complete segregation with the phenotype in the family; (3) as predicted, there was no detectable level of MICU1 protein in the patient fibroblasts; and (4) this caused an anticipated defect of mitochondrial calcium handling, which was rescued by expressing the wild-type protein.

The calcium uptake rate difference, which is reminiscent of *Micu1* knockdown in mouse liver mitochondria,^9^ has 2 possible explanations. MICU1 has been proposed to be an activator of the uniporter, in which case removing it could result in reduced calcium uptake rate.^7,10^ Alternatively, MICU1 has also been proposed to be a gatekeeper of the uniporter that prevents baseline levels of cytosolic calcium from entering into mitochondria.^6–8^ In this case, the observed lower calcium uptake rate may actually represent a secondary consequence of increased basal matrix calcium in the absence of MICU1, which would reduce the driving force for calcium uptake. This is consistent with what has previously been reported in skin fibroblasts from patients lacking *MICU1*.^11^ Consistent with the latter model in which cells have higher basal matrix calcium, the levels of phosphorylation of pyruvate dehydrogenase (PDH) at S293 are higher in the homozygous fibroblasts (figure 3, F and G). The PDH phosphatase is activated by matrix calcium, leading to dephosphorylated (activated) PDH. Thus, decreased PDH phosphorylation would be consistent with increased matrix calcium levels. As expected, both CCCP and oligomycin reduced PDH phosphorylation, whereas serum starvation resulted in increased PDH phosphorylation, showing that the assay could detect both increases and decreases in PDH phosphorylation.^12^

Numerous hurdles obscured the diagnosis in this family. For several years the nonspecific clinical course precluded specialist referral. Further investigation followed a detailed family history and the CK measurement. This combination of fluctuating fatigue, migraine with obtundation, and a high CK level raised the possibility of a mitochondrial disorder, but conventional respiratory chain studies were normal. The diagnosis became apparent only by analyzing the exome sequence read depth. Based on the limited clinical descriptions published to date, our patients have a similar phenotype to patients with *MICU1* substitutions, although cataracts have not been noted before (table e-1, reference 11).

The extreme clinical fluctuation described here is reminiscent of an ion channelopathy. The likely role of MICU1 as a calcium-sensing regulator of the mitochondrial calcium uniporter^7,13^ provides an explanation for the fluctuating clinical course seen in patients with *MICU1* and raises the hypothesis that calcium channel blockers may alter the disease course.

## Supplementary Material

Data Supplement

## GLOSSARY

CK: creatine kinase
GATK: Genome Analysis Toolkit
MAF: minor allele frequency
PDH: pyruvate dehydrogenase
SNVs: single nucleotide variants
