# Supplementary material for: Homozygous deletion in MICU1 presenting with fatigue and lethargy in childhood
Source: Neurol Genet. 2016 Mar 3;2(2):e59. doi: 10.1212/NXG.0000000000000059 (PMC4830195; doi:10.1212/NXG.0000000000000059)
Supplement: Data Supplement [file supp_2_2_e59__index.html]

Data Supplement 

# Homozygous deletion in *MICU1* presenting with fatigue and lethargy in childhood

## Data Supplement

**Files in this Data Supplement:**

- Table e-1 - Microsoft Word file
