# Supplementary material for: Homozygous deletion in MICU1 presenting with fatigue and lethargy in childhood
Source: Neurol Genet. 2016 Mar 3;2(2):e59. doi: 10.1212/NXG.0000000000000059 (PMC4830195; doi:10.1212/NXG.0000000000000059)
Supplement: Data Supplement [file supp_2.2.e59_Table-e-1.docx]

**Table e-1**. Shared single nucleotide variants and short insertion/deletions identified by exome sequencing the two cousins IV:3 and IV:6.
